# A Novel Mathematical Method to Diagnose the Transverse Growth Deficit of the Nasomaxillary Complex

**DOI:** 10.3390/diagnostics12071537

**Published:** 2022-06-24

**Authors:** Clara Guinot-Barona, Inmaculada Soler Segarra, Santiago Arias de Luxán, Raquel Laparra Hernández, Laura Marqués Martínez, Esther García Miralles

**Affiliations:** 1 Faculty of Medicine and Health Sciences, Catholic University of Valencia, 46001 Valencia, Spain; clara.guinot@ucv.es (C.G.-B.); esther.garcia@ucv.es (E.G.M.); 2 Department of Dental Medicine, Faculty of Medicine and Dentistry, University of Valencia, 46001 Valencia, Spain; inmaculada.soler@uv.es; 3 Orthodontics, Dentistry Department, Faculty of Medicine and Health Sciences, University CEU-Cardenal Herrera, C/Del Pozo s/n, Alfara del Patriarca, 46115 Valencia, Spain; santiago.arias@uchceu.es (S.A.d.L.); raklae@gmail.com (R.L.H.)

**Keywords:** crossbite, skeletal compression, transversal malocclusion, diagnostic mathematical method

## Abstract

The diagnosis of transverse growth deficit of the maxilla in daily clinical practice is carried out mainly through the experience of a well-trained clinician, which implies a lack of objective criteria applicable in a protocolized manner. The objective of this study was to establish a mathematical method to diagnose maxillary compression in relation to the dimensions of the skull and mandible. Methods: Records of 97 cases with an overall mean age of 9.8 ± 2.6 years were analyzed by three experienced orthodontists. The group of transverse compression was comprised of 62 cases and the control group of 35 cases. The main measurements of the widths were made on a frontal teleradiography of the skull (cranial, zygomatic, orbital, maxillary, bigonial and biantegonial width) and a lateral teleradiography of the skull (facial axis, mandibular plane, SNA, SNB, ANB and Wits). It was established that from the cranial width it is possible to predict the group to which each subject studied belongs—the compression group or the control group. A mathematical formula was obtained in the form of logistic regression that allows for the diagnosis of the presence of maxillary compression based on the cranial, maxillary and orbital widths with a sensitivity of 88.7% and a specificity of 77.1%.

## 1. Introduction

Malocclusion problems in the horizontal or transverse plane are considered an important clinical entity. On many occasions, they are associated with the presence of a posterior crossbite and/or a scissor bite, which in turn can generate a lateral slippage of the mandible, which, if not corrected, will lead to skeletal asymmetry [1].

However, it is also possible to find disproportion at the skeletal level between the upper and lower jaws which can be compensated dentoalveolary by the buccal-lingual inclination of the upper and lower molars, without, therefore, any dental alterations in the transversal plane [1].

The transverse growth deficit of the nasomaxillary complex is also named in the literature with different terms such as skeletal compression of the upper jaw or transversal maxillary hypoplasia. This deformity constitutes a clinical entity encompassed within malocclusions or transversal dysmorphia. In such a way that an alteration of the skeletal relationship between the width of the apical bases of the upper and lower jaws is manifested, the origin of said alteration being either a defect of the upper jaw or the lower arch, both at the dental and skeletal level are absolutely normal [1,2].

The frequency of occurrence of transversal problems originating in a deficit of the upper jaw varies slightly between different authors; usually, its prevalence is around 10–23% in the general population [3]. These values are higher if all patients presenting a transverse maxillary problem without the association to functional alterations are included [1,2,4].

The etiopathogenesis of the maxillary transversal deficit is multifactorial, including congenital malformations, developmental, traumatic, and iatrogenic abnormalities [5]. In addition, environmental factors are also added, highlighting the important role of harmful oral habits within this framework: oral breathing, infant swallowing and digital sucking [5,6].

### Diagnosis in the Transverse Plane

Diagnosis of the maxillary transverse developmental deficit has been made mainly through the clinical judgment of the professional. The first method that established a series of standards for the topographic diagnosis of transverse problems dates back to 1981, almost 50 years after the introduction of cephalometry by Broadbent, initially in the United States and Germany [7].

Neither the use of plaster models nor diagnosis solely at the clinical level by examining the patient is considered an adequate method to assess the problem in the transverse plane [8]. Only including a detailed transverse plane evaluation of the structures of the skull and face will lead to a complete analysis of the dentofacial problem [9].

The great milestone in frontal teleradiography began with the studies of Broadbent, around 1930, where he exposed the main cephalometric findings that he collected based on the normal growth of the face. This allowed the development of different analysis methods for such a type of projection [7]. Authors as important as Ricketts, Brodie, Downs, Tweed, Björk and Steiner developed the most popular analyses of this type of cranial radiography [10]. However, despite the great advantages that these studies offer, and the fact that they are the only ones that allow the existence of real facial asymmetries to be assessed and quantified, their use is not very practice as a radiological image projection that is often not included in the daily routine of orthodontic offices [11]. 2D radiography has undergone a revolution in recent years with the use of CBCT (Cone Beam Computed Tomography). Its use has been introduced in orthodontics as a technique for comprehensive orthodontic imaging for the assessment of clinical cases, involving skeletal and dental frames, in all three planes. Although it brings important benefits, one major concern regarding CBCT is the higher radiation dose delivered from CBCT scanners with a large FOV. Therefore, the decision to conduct CBCT imaging on an orthodontic patient should be made individually only when CBCT is required for proper diagnosis and an optimal treatment outcome, and 2D records should continue to be used in routine examination [12].

One of the most common errors during the study of transverse problems is to compare their diagnosis to the presence or absence of crossbite at the oral level. This commonly generates a functional deviation of the mandible, aggravating the underlying skeletal problem. This deviation in turn generates a facial asymmetry that derives from a deviation in the position of the condyles [5].

Such mandibular deviation of the great functional component in many cases resolves spontaneously only with the deprogramming of the patient, thus reaching a spontaneous centering of the mandible. It is essential to highlight that the sooner the problem is corrected, the greater the functional component will be represented and the less the skeletal, so a complete correction of the mandibular position may even occur. If, on the other hand, the correction of the problem is delayed until late puberty, the skeletal component will exceed the functional one, leaving, once the crossbite has been corrected, a true skeletal deviation that is often noticeable facially [5].

McNamara (2002), one of the authors who has studied the most on this topic, highlighted that until nowadays, the treatment of rapid maxillary disjunction had been applied almost exclusively to cross bite cases. However, in recent years it has begun to be used more extensively in those cases in which the existence of maxillary skeletal compression was considered without any dental alterations, such as crossbites. Its use has been extended to the following cases: cases with reduced intermolar width, considering a width between the upper first molars at the level of the mesial-palatine cuspids of less than 31 mm, with or without crossbite; cases with an exaggerated Wilson curve; cases with important wide buccal corridors due to an extremely narrow arch, which benefit from this type of treatment in an attempt to widen the smile arch and cases where a moderate bone-dental discrepancy is observed, in which the widening of the upper jaw would improve not only the upper crowding directly, but also indirectly by acting on the resolution of the lower crowding. This phenomenon is mainly due to the fact that despite direct actions on the width of the mandible cannot be carried out, the expansion of the upper arch will lead to a straightening of the lower teeth, improving the coronal inclination that will result in an increase of the perimeter of the dental arch. More controversial cases are those in which a respiratory deficit is present. Mainly in milder cases of obstructive sleep apnea syndrome in children, in which the indication for surgical treatment is not clear, skeletal expansion of the maxilla could improve respiratory capacity by widening the nasal cavity. Finally, the main application of this type of treatment is for the presence of skeletal class III, which is due in the vast majority of cases to a retrusion of the upper jaw rather than to a true protrusion of the mandible, usually accompanied by a reduction in the transverse diameter of the maxilla [13].

The purpose of the present work was to establish a new diagnostic protocol for the transverse growth deficit of the maxilla, in the form of logistic regression, throughout the analysis of the maxillo-cranial and maxillo-orbital complex of the frontal radiograph.

## 2. Materials and Methods

To carry out the present study, the clinical records of the database of a Dental Clinic in Valencia (M-A Clinic) were analyzed and those that met the following inclusion criteria were selected: lateral teleradiography of the skull and frontal teleradiography of the skull in the anteroposterior projection of healthy patients aged 5 to 18 years with transverse problems. Cases in which the radiographs showed dental absences not typical of age or in which lateral or frontal teleradiography of the skull did not allow for correct location of the points either due to lack of cooperation of the patient or an incorrect technique were excluded.

All cases were re-evaluated by 3 orthodontic specialists with more than 10 years of experience in the field of orthodontics with the aim of classifying patients into 3 groups: Group 1 (growth subjects suffering from a transverse maxillary deficit), Group 2 (subjects without transverse problems) and Group 3 (cases in which the orthodontic specialist did not have a clear diagnosis, leaving the decision of whether to carry out a skeletal expansion treatment of the upper jaw based on other variables such as the position of the upper canines, the amount of crowding present in the arch, or a family history of maxillary compression).

The final sample of the study was constituted of those cases where at least 2 of the 3 experts had classified the case within the same category, obtaining a final sample of 97 cases—62 cases in Group 1 and 35 cases in Group 2—with an overall mean age of 9.8 ± 2.6 years and a range between 5.5 and 17.8 years and a median of 9.3 years.

**Figure 1:**
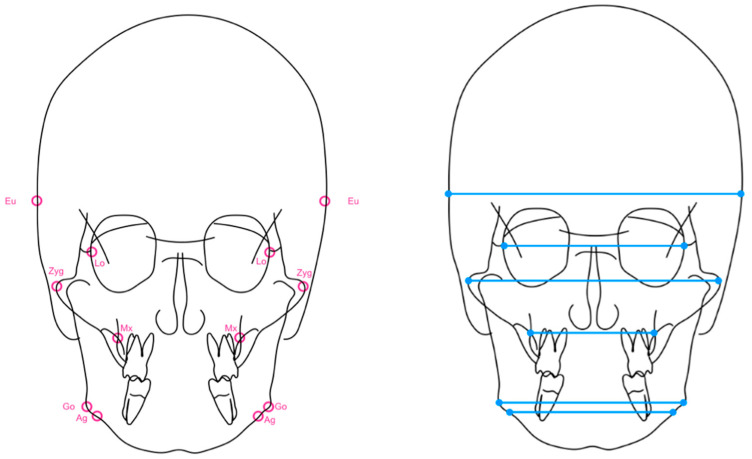
Representation of the cephalometric points studied in the frontal teleradiography and planes analyzed: Eu (Euryon) is a bilateral point that corresponds to the most lateral point of the cranial vault. Lo (Latero-orbitale) is a bilateral point generated by the intersection of the lateral wall of the orbit and the greater wing of the sphenoid. Zyg (Zy gomatic) topographically corresponds to the most lateral aspect of the zygomatic arch bilaterally. Mx (Maxillare) is a bilateral cephalometric point obtained by the intersection of the external line of the maxillary tuberosity and the zygomatic process. Go (Gonion) is a bilateral measurement defined as the point located at the gonial angle of the mandible. Ag (Ante-gonion) is a bilateral point located at the level of the antegonial curvature at the maximum curvature point.

An analysis of the cases was carried out where the following data was collected:

- Facial axis, mandibular plane, ANB and Wits.
- Maxillary and mandibular width: frontal teleradiograph of the skull was measured using the Kodak Dental Imaging Software 6.12.11.0 computer program, converting the automatically generated magnification to a 1:1 image ratio, with the aim of standardizing the magnification produced. Cranial width, orbital width, width between the zygomatic bones, maxillary width, and mandibular width were obtained (Figure 1).
- Difference between maxillary and mandibular width, difference between orbital and maxillary width, difference between zygomatic and maxillary width, difference between maxillary and bigonial width and difference between maxillary and biantegonial width.
- Chronological age.
- Biological age, according to the Demirjian method [14].

### Statistical Analysis

Statistical analysis was performed using the SPSS version 15.0 program, with a significance level of 5% (α = 0.05) and a confidence value of 95%, where homogeneity was assessed using the *t*-test for independent samples and with normality fit (confirmed by the Kolmogorov–Smirnov test). Descriptive and inferential analysis of the data collected was performed using the chi-squared test and a binary logistic regression model was estimated to explain/predict the diagnostic group from each of the cephalometric variables. The odds ratio (OD) of the unadjusted association and 95% confidence intervals were provided. Next, a model was made, also logistic, but in this case multiple, with the set of independent variables. The objective of this analysis was to identify the optimal subgroup of variables (determining as optimal variables those that could best explain the presence of maxillary compression) and to consider that any other does not increase the explanatory capacity of the model.

Data analysis was performed using simple models and multivariate models: sensitivity, specificity, and the predictive value of positive and negative tests. The ROC curve (Receiver Operating Characteristic) was also obtained for the diagnostic rule, thus evaluating its discriminatory capacity by estimating the AUC (Area Under the Curve). The typical error was also provided, with a confidence interval at 95% of the AUC and a contrast test AUC of 0.5.

Finally, the model was re-estimated on an estimation sample corresponding to 60% of the cases of the global sample and it was evaluated on the remaining validation sample. Validity parameters were recomputed to compare the potential loss of efficacy and method error calculation was performed.

## 3. Results

**Figure 2:**
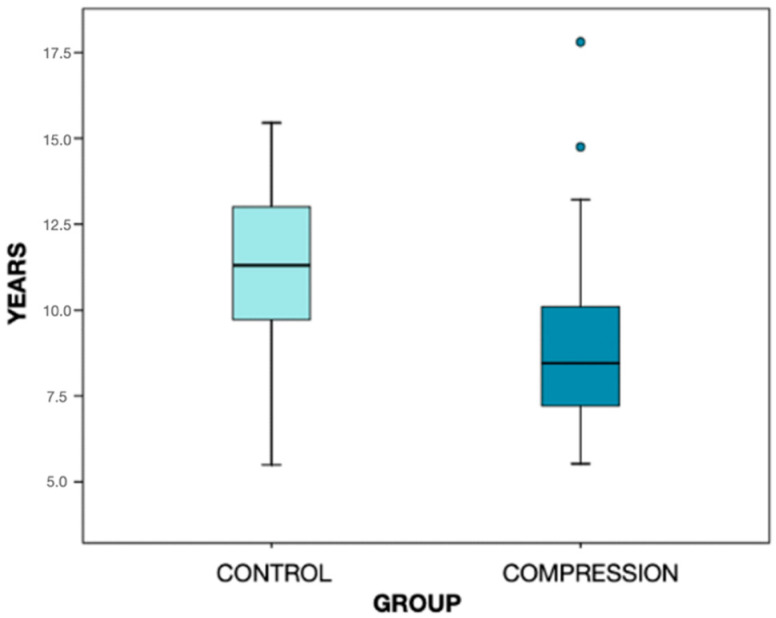
Graphic representation of the distribution by age of the two groups analyzed. The box contains 50% of the cases, being the horizontal divisor line the median. The mean age of Group 1 is 8.9 ± 2.3 years and the mean age of Group 2 is 11.3 ± 2.5 years. The two plot points in the compression group correspond to the outliers, that differ significantly from the rest of the dataset.

The total number of the sample was 97 subjects with an overall mean age of 9.8 ± 2.6 years, with a range between 5.5 and 17.8 years and a median of 9.3 years. Group 1, or the transverse compression group, was comprised of 62 cases of which women accounted for 53.2%, and Group 2, or the control group, had 35 cases, of which 42.9% were women (Figure 2).

### 3.1. Descriptive Analysis

#### 3.1.1. Lateral Teleradiograph of the Skull

The measurements of the variables of the lateral skull radiograph were analyzed as a whole as an absolute value, without carrying out a classification of each one of the cases independently, in an attempt to observe the general trend of the variable in the group. It must be highlighted that the median value of the facial axis was similar in both groups with a value of 89°, which shows growth close to facial balance, with a similar minimum of around 81° and a maximum of 100°. In relation to the mandibular plane, whose balanced value is 32°, a median of 33° and 35.5° in the control and study groups, respectively was found.

When assessing the relationship between the maxilla and the mandible, an ANB was detected with a median of 3° in the compression group and 4° in the control group, both values within skeletal normality. However, it is noteworthy that in the compression group, the minimum and maximum values showed greater extremes of −5° and 10°. This ends in the inclusion of much more extreme cases of skeletal class III and II in the compression group. Finally, when analyzing the Wits value, a median with a negative value (−0.5) is observed, which, even when included within a class I balance, shows a tendency towards class III.

#### 3.1.2. Frontal Teleradiograph of the Skull

Among the results obtained from the frontal teleradiograph of the skull, the following findings stand out:

The cranial width value showed a median of 131.60 mm in the compression group and 126.00 mm in the control group. The value of the orbital width also showed a higher median of 86.40 mm in the compression group compared to 78.90 mm recorded in the control group. This same phenomenon was observed in the zygomatic width, although with smaller differences: 109.85 mm in the compression group and 107.90 mm in the control group.

When analyzing the data collected regarding maxillary width, similar median values are observed which are slightly higher in the case of the control group: 58.90 mm compared to 57.60 mm in the compression group.

It stands out, therefore, that in the face of larger measurements of cranial dimension, lower values of maxillary width were detected in the group diagnosed with a transverse growth deficit of the maxilla. Values regarding mandibular width showed very similar results with respect to the median in both groups, both measured at the Ag point and the Go point.

Thus, from a descriptive analysis of the sample, the most striking findings are the differences in the upper facial third between both study groups.

### 3.2. Statistical Analysis Based on the Direct Variables Obtained in the Lateral and Frontal Teleradiograph of the Skull

Data obtained from the lateral skull cephalometry were studied in order to evaluate the skeletal class and the facial pattern. (Table 1 and Table 2).

Within the linear measurement group, cranial width, orbital width, and Wits showed statistically significant differences between the two groups.

Cranial width and orbital width showed higher values in the compression group. The Wits index, which determines the patient’s skeletal class, has a negative mean of −0.15 ± 2.79 in diagnosed subjects compared to 1.06 ± 2.43 in control group subjects, showing a trend towards skeletal class III. The zygomatic width, another of the variables analyzed, showed a strong tendency to present higher values in the group of case patients.

Likewise, within the angular measurements, the MD plane and the SNA value were the most heterogeneous angular parameters between the study groups, also exhibiting a strong statistical tendency to be higher and lower, respectively, in the group of case patients in relation to the group of patients with compression.

### 3.3. Data Analysis Using Simple and Multivariate Models

#### 3.3.1. Simple Diagnostic Discriminant Models of Absolute Variables

From the results shown above, a first view was obtained in which variables showed differences between the two study groups and, therefore, could have a certain prognostic value in the diagnostic decision to classify a case as healthy or with compression.

Using such information, a model was created in which “*p*” was defined as the probability that a subject presented the anomaly in question. Specifically, a variable derived from “*p*” was used, the so-called odds *p*/(1 − *p*), which is related through a logistic regression model with each of the independent variables of the study.

**Figure 3:**
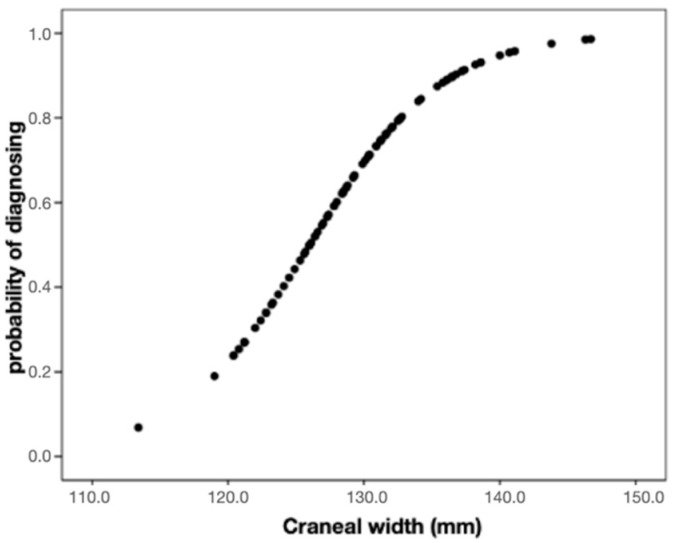
The graph allows the visualization of the increase in the probability of diagnosing maxillary compression as the width of the skull increases.

It was observed that the cranial width significantly increased (*p* < 0.001) the probability that the individual would be classified in the maxillary compression group. Specifically, for each additional 1 mm of width, the odds (or risk) of presenting the diagnosis is multiplied by 1.23, thus rising by 23% (Figure 3).

Therefore, it was established that from the cranial width it is possible to predict the group to which each subject studied belongs, the compression group or the control group.

Once the impact of each of the variables on the probability of having the deformity was assessed, the number of patients who were correctly classified in the sample from the model was studied.

To assess the quality of the predictive model, the sensitivity, specificity, and positive and negative predictive value of the application of the formula on the initial sample were calculated, assessing the number of patients that were correctly classified by applying the formula. Of the 62 subjects with compression, a model like this (unadjusted, based exclusively on the reading of the cranial width) will be correct, detecting the true positives in 87.1% of the cases (this is the sensitivity). On the other hand, among the controls, 54.3% will be classified correctly as being without compression, that is, as true negatives (this is the specificity). Globally, it is stated that 75.3% of individuals are correctly classified.

This analysis procedure was repeated for all measured variables as shown in Table 3.

The findings obtained allow us to establish that: Zygomatic width exhibits an effect on diagnosis close to statistical significance (*p* = 0.071). Specifically, an additional 1 mm of this length implies a 7.5% increase in the probability of a diagnosis of compression. A model based on the zygomatic width would have a global percentage of correct identification of our case patient of 64.9%. Orbital width is another key measure to diagnose: the association is significant (*p* < 0.001), estimating an OR = 1.52, that is, each additional mm of orbital width implies a 52% more probability of compression.

If a logistic equation based on this parameter is established, the subjects of the compression group will be correctly classified 80.4% of the time. The sensitivity of the predictive rule will be 87.1% and the specificity 68.76%. If the measurements that showed a strong tendency are analyzed, the mandibular plane stands out. The impact of changes in this angle on the odds of compression is moderate (OR = 1.076); but the estimated function perfectly reflects the clear existence of an association. A model-based exclusively on this measure can be considered correct at a moderate level: 64.9%, partly due to its high sensitivity at the cost of very poor specificity (most patients are predicted as affected).

In the case of the ANS, a similar situation was observed, even weaker than the previous one (*p* = 0.089). If SNA increases, the probability of compression decreases, but in this case, the degree of accuracy in the diagnosis based on this value will be considered very low, 62.9%.

Finally, if the Wits value is assessed, it is observed that the estimate obtained suggests that 1 more mm of Wits imposes a compression risk reduction of 16.2% (OR = 0.84).

#### 3.3.2. Multivariate Models of Absolute Variables

Once the impact of each of the parameters had been explored separately (unadjusted models), the estimation of a complete or multiple models was proposed, which would allow for improving the positive predictive value and, therefore, the ability to detect the subjects with compression as such, including the values whose variation was statistically significant in the compression group and adding maxillary width, previously without relevance (Table 4).

It is important to highlight that these variables that were previously significant were not included in this model, not because when they were introduced they did not have a statistical relationship, but because in the presence of the previous three variables (the cranial, orbital and maxillary width), they did not provide novel results. Thus, despite complicating the regression model, they did not increase the predictive capacity of the calculated diagnostic model and, therefore, were excluded.

Based on the model obtained from the results, it can be stated that in the calculated diagnostic model, the cranial width significantly increases (*p* = 0.007) the probability that the individual presents maxillary compression. For each additional 1 mm of width, the odds (or risk) of presenting the diagnosis is multiplied by 1.18 (the probability increases by 18%). The orbital width significantly increases (*p* < 0.001) the probability of diagnosing a subject with maxillary compression. For each additional 1 mm of width, the odds (or risk) of presenting the diagnosis is multiplied by 1.76 (the probability increases by 76%). Finally, maxillary width reduces the risk of classifying a patient with compression. It does so in a percentage of 31% (OR = 0.69) for each additional 1 mm in length.

To assess the fit of the model to the data, the Nagelkerke R2 coefficient was calculated: in this case, it is 0.682, which is moderately acceptable in terms of goodness of fit. On the other hand, the Hosmer–Lemeshow test was also found, which allows us to accept the hypothesis that the model fits adequately (*p* = 0.925).

When applying this model to the total sample, it can be stated that of the 62 cases with the alteration in the sample, the calculated model is capable of being correct in 55 of them, which means a sensitivity of 88.7%. Of the 35 patients classified as controls, the model is able to correctly predict the absence of pathology in 27 (77.1%). These results translate to a total of 82 well-classified cases (84.5%). From these results, the positive predictive value and the negative predictive value can also be calculated: the model is capable of predicting compression in 63 cases and is correct in 55 (PPV = 87.3%). In addition, it is capable of predicting the situation of no compression in 34 cases and correct in 27 (PPN = 79.4%).

The equation of the logistic model responds to the following expression:

(1) $$\frac{p}{1-p}=3.7\times 10^{-21}\times 1.18^{\mathrm{craneal}}1.76^{\mathrm{orbital}}0.69^{\mathrm{maxillar}}$$

When reading the model, “*p*/(1 − *p*)” will be calculated, where “*p*” is defined as the probability that a subject presents a higher transversal deficit, and, clearing the formula, we obtain *p*.

If, for example, the entire expression on the right is called “a”, it would be observed that “*p*/(1 − *p*) = a”, therefore “*p* = a/(1 + a)”. Given a certain cranial, orbital and maxillary width of a subject, it is substituted into the formula and the predicted probability ‘*p*’ of having compression is calculated so that if *p* > 0.5, the case will be predicted as with compression, and if *p* < 0.5, the case will be predicted as control-no compression.

### 3.4. Evaluation of the Model of Absolute Variables in an External Sample

The equation obtained comes from the total data available in the study (the 97 patients in the total sample). Obviously, its predictive capacity will be maximum when it is applied to the same sample, as has been performed in this study.

The true predictive power of a model, such as the one designed, is achieved by evaluating its results in an external sample, different from the current one but from the same population.

Taking into account the fact that the current sample size is considered large, it can be divided into two parts in proportions of approximately 60% and 40%. The purpose of doing it this way is that the largest part (60% of the total) serves to re-estimate the model again and the smallest (40% of the total) allows to check its degree of success. The main issue will be to study how this new formula would behave in the set of 40% that constitutes the “validation sample” (Table 5).

In order to understand this table, we can say that of the 23 severely affected cases in the sample, the model is able to hit 78.3% of the cases (S) and of the 19 healthy ones, it hit 89.5% (E) of the cases. Thus, the classification is correct for 35 individuals, which constitutes 83.3% of the total. In other words, the model has a similar predictive capacity. It is expected that in future samples (of sufficient size) it will work with the same level of success, concluding that the sample size of our group was adequate (Table 6).

## 4. Discussion

Prior to planning and designing this study, the need to carry out this diagnostic study was raised, for which a search of the literature was performed to establish the measurement methods that had been used on frontal teleradiographs for the diagnosis of maxillary compression deformity. Regarding the results obtained, the lack of analysis of this type of image was corroborated, especially given the multitude of existing methods for assessing the problems that occur in the anteroposterior plane, as stated by Athanasiou et al. [15].

Regarding the design of a logistic regression model, no similar study was found in the literature, based on the analysis of frontal teleradiographs of the skull, with the aim of performing a multivariate model to obtain an easily applicable diagnostic tool in the daily clinic.

The first to describe a method to quantify the transverse problem at the maxillary level was Ricketts (1981) through the study of frontal teleradiographs of the skull. Although it is true that the introduction of this image projection was much earlier (almost 50 years earlier), it was not until 1968 that it began to be used in studies on the growth and development of human beings. The introduction of skeletal and/or dentoalveolar expanders of the upper jaw expanded new horizons in orthodontic practice, forcing the need to establish specific standards capable of quantifying the discrepancy between maxillary and mandibular width [16].

When analyzing the evaluation of the methods used, all the radiographs were obtained from the same office and were measured with the same computer program. The analysis obtained a high degree of reproducibility, so the digital measurement and location of the cephalometric points used turned out to be an adequate and reliable procedure.

If the most frequent dentoalveolar characteristics and traits that can be found both in patients with a transverse growth deficit of the nasomaxillary complex and in patients with normal transverse dimensions are analyzed, a greater number of studies and data in the analyzed literature are determined. Yavuz et al. [9] found that the cranial width is a practically constant value in development from 6 years of age, with slightly more development remaining in the facial, nasal, maxillary and mandibular width. These same authors agree with Graber et al. [17] in the sequence of development, according to which the transverse dimensions are much more stable and stop before the anteroposterior and vertical ones.

The study found that one of the variables most closely related to the presence of superior skeletal compression was cranial width and its difference in relation to the maxilla. If it is taken into account that it is one of the most stable factors from the age of 6, it can be affirmed that it is definitively related to the diagnosis of the deformity, regardless of the age of the subject.

When analyzing the data obtained in the group of cases from the lateral projections, it is observed that the only statistically significant measure in relation to the healthy group is the Wits, which shows a tendency to a negative result according to the mean obtained (−0.15 ± 2.79), which shows a tendency of the transverse growth deficit of the maxilla and skeletal class I borderline towards class III.

Within the reviewed literature, only one study was found that analyzed the radiological characteristics at the frontal level in class III in the geographical environment in which the present investigation was carried out [18]. These same authors highlight the absence of more cross-sectional studies on this skeletal malocclusion as the result of the extensive application of the treatment protocol for this type of deformity, consisting of the use of a skeletal expansion of the upper jaw prior to the anterior traction of the maxilla, either through extraoral appliances (a face mask) or through the use of mini plates and/or mini screws. It is then stated that the need to carry out a cross-sectional study of this type of case has been relegated to the background for years. In conclusion, since the effectiveness of anterior traction improves with expansion, it is not important to study whether expansion is necessary or not due to an underlying transverse problem, because regardless of the result obtained in the analysis in the frontal plane, the maxilla should be expanded for a more successful result.

Performing the first study based on a posteroanterior skull radiograph, Franchi and Baccetti [18] found that the maxillary width was 3.8 mm smaller in class III cases when compared to skeletal class I. This same procedure was also performed for the skeletal class II group, where a difference in maxillary width also appeared, but of a smaller dimension than in the previous case (2.5 mm smaller). If these results are compared with those obtained, a concordance is observed, since the Wits value together with the standard deviation falls within the normal values of skeletal class I as well as class III and light class II [19].

In addition to the study already mentioned [19], two others analyze the transverse characteristics at the radiological level of class II and class I cases. Alarashi et al. [20] described the characteristics in the frontal plane on a teleradiography of the skull in cases of class I and II first division (in which a proinclination of the upper incisors is characteristic) without an apparent transverse problem. The need to assess the transverse malocclusion in an X-ray is highlighted, especially at greater discrepancies at the anteroposterior level. These authors found statistically significant differences in maxillary width and upper intermolar width, these being narrower in class II/1 cases than in class I cases. These results were in turn associated, although not statistically significantly, with greater vertical growth of the upper jaw, more typical of dolichofacial patterns. A certain contraction of the nasal width was also shown, but it was not statistically relevant. However, no differences in mandibular width were shown when comparing class I and skeletal class II cases. When comparing these data with the results obtained, it can be observed how subjects who present a greater transverse defect of the maxillofacial complex showed higher maximum values in terms of anteroposterior retrusion of the upper jaw (ANS), normally associated with class III, and minimum values of mandibular position (SNB) more extreme than the controls, associated in this case with class II.

Lux et al. [21] also analyzed the transverse cephalometric characteristics of classes I and II, including in this case both a subgroup of class II/1 and class II/2. They found that class II cases with maxillary incisor proclination had narrower jaws than any other group. No statistically significant differences could be found in mandibular size between the groups (C I, C II/1 and C II/2). This coincides with the results of the research carried out, according to which the mandibular width is not a significant datum to take into account when assessing the presence of a transverse growth deficit of the upper jaw. However, in the design of the study, a great difference is found with respect to the one developed in the present work and the two found in the literature, since the frontal teleradiographs were taken with an inclination of 35° with respect to the Frankfurt plane, so they are not comparable.

In addition to what has been said so far, it can be observed how the analyzed literature shows that any skeletal class can be associated with a transverse growth deficit of the maxilla, both in extreme cases of class III and cases of mandibular retrusion and class II.

This absence of a relationship between the skeletal compression of the upper jaw and a clear skeletal class is also shown in the present study, as there is no statistically significant relationship between the case group sample and the result of the ANB variable, despite the fact that there is a tendency toward negative values of the Wits index.

This is a key point to highlight since when designing the study and selecting which variables should be analyzed, two assessments were included for each of the characteristics studied. Thus, on the one hand, the skeletal class was defined by the Wits and ANB values and, on the other hand, the facial growth pattern was defined by the facial axis and the mandibular plane. In this way, it was possible to verify or rule out a possible statistically significant result. This same phenomenon was verified again with the analysis of facial growth. In the statistical study of independent variables, there was a certain statistical tendency of the mandibular plane to be greater in subjects from the compression group (36.2° compared to 33.8° on average in the healthy group), which could have shown a certain tendency toward vertical growth of subjects with compression. However, when checking this suggested relationship in light of the results obtained with the second variable analyzed on vertical growth, it was observed that the angle of the facial axis did not show any statistical relationship between the groups. Therefore, such an association was ruled out as statistically significant but left a statistical suspicion between the more vertical patterns and the presence of a skeletal compression deficit of the maxilla.

This requirement of double assessment of the skeletal class and the facial pattern made it difficult to classify the cases. Initially, the design was planned with the idea of dividing the sample into three subgroups according to facial pattern and skeletal class. However, in most cases, one of the values classified the subject in one group and the second variable determined the classification of the same subject in another group. Thus, for example, for a specific patient, the ANB could be within the class I parameters, while the Wits belonged to the class III set. For this reason, it was finally decided to consider only the absolute values of the variables as well as the measures derived from them (mean, median, maximum and minimum).

In the literature, based on the analysis of the cross-sectional characteristics based on diagnostic models, it is detected that patients who show a class II/1 dental relationship manifest a smaller dimension of the dental width of the upper arch [22].

Coinciding with these same authors, Bichara et al. [23] described how the subjects with this dental malocclusion showed this relative compression from the first stages of primary dentition until the dental replacement was completed, validating the premise already stated that there can be no relationship between the age of the patient and the probability of the transverse problem of the maxilla, as the results obtained could appear a priori; on the contrary, the deficient width of the maxilla not only does not resolve spontaneously but also worsens over time. DaSilva et al. [24] agree with these same authors when studying the dental characteristics of dental class II, thus recommending an assessment of the need to perform an expansion of the upper arch prior to sagittal correction. They did not establish whether this expansion must be skeletal or dental, since they did not analyze radiographic records and, therefore, did not take into account the width of the skeletal bases.

Sayin and Turkkahraman [25] designed a study using plaster models to assess not only tooth width but also dentoalveolar width, measuring the greatest width of the model at the vestibular level in class II first division subjects and class I subjects. They concluded that there is indeed a tendency to compress the upper dental arch in the case of subjects with distocclusion, but that these differences dissipate when measured at the level of the dentoalveolar process (as they call it). Despite coinciding with the results of the study carried out (in which a statistical relationship between skeletal class and the presence of maxillary compression could not be established), this study method raises great doubts because it is not corroborated a frontal projection of the skull that makes it possible to demonstrate the findings that were observed.

The only one of the studies analyzed that makes an exclusive reference to the dental characteristics of class II/2 cases [26] shows that this type of malocclusion presents more similar characteristics with respect to classes I than to classes II/1, with broader dental arches, without apparent constriction. The slight compression that appears at the level of the lower arch stands out exclusively, probably due to the effect caused by the overbite typical of this type of malocclusion, by totally blocking the lower dental arch (this reduction in the dental diameter being more pronounced at the level of the canines).

Therefore, it can be concluded that neither the predominant growth pattern in the patient nor the skeletal class shows a statistically significant relationship with the presentation of the transverse growth deficit of the maxilla, and it is not possible to identify a specific pattern.

Regarding the analysis of the relationship between the gender variable and the presence of a transverse growth deficit of the maxilla, no statistically significant relationship is found in the literature regarding this premise. In our results, a concordance is detected with respect to this condition, since, although women accounted for a higher percentage of patients than men in the case group, these data were not statistically significant.

Finally, if the results obtained through the individual analysis of the linear variables and the multivariate analysis carried out on the relationship established between the maxilla and the rest of the linear measurements obtained in the frontal teleradiograph of the skull are evaluated, it is observed that at first Ricketts (1981) exclusively related the width of the maxilla with the width of the mandible, regardless of the rest of the cranial morphology, with the only differentiation between sex, thus attending to the different development times experienced by men and women respectively. If only the studies obtained that can be compared to this method are taken into account, the study by Lux et al. should be discarded. [20] since the imaging technique differs enormously in relation to the other three articles studied, which placed the Frankfurt plane parallel to the ground.

Therefore, only the studies by Franchi and Baccetti [18] and Nanda et al. [27] were comparable. None of the studies tried to extract a diagnostic method for this type of dentofacial deformity; the former extracted prediction tables for the normal growth of the nasomaxillary complex, based on absolute values without establishing proportional relationships, which are of much more interest, taking into account the wide differences that may exist in the growth and development of a sample group of the same age as a whole, but whose cranial complexion characteristics differ radically. The second group of authors determined that class III and class II cases show a certain tendency towards maxillary compression, especially those of class III. This fact could not be correctly contrasted in the present study since the sample size was insufficient and lacked clear criteria that would allow dividing the cohort into three groups according to skeletal class. However, certain minimum and maximum values were observed in the maxillary compression group, so it is possible to detect a certain tendency to associate the most extreme anteroposterior discrepancies with the transverse growth deficit of the maxilla.

## 5. Conclusions

Based on the obtained findings, it can be concluded that:

- The new mathematical method presented based on the cranial, orbital and maxillary width, with high sensitivity values, is capable of diagnosing cases of skeletal compression of the upper jaw.
- In the sample studied, the probability that a case presents a transverse growth deficit of the nasomaxillary complex through the association of the maxillo-cranial and maxillo-orbital difference can be determined numerically with a sensitivity of 88.7% and a specificity of 77.1%, a positive predictive value of 87.3% and a negative predictive value of 79.4%.

There are also some limitations of this study. Obtaining a greater number of records by age group would allow each age group to be evaluated independently. In addition, using the model of a frontal cortex obtained from a CBCT would allow verification of the method in this type of projection, which is the most widespread today.

## Figures and Tables

**Table 1:** Homogeneity of cephalometric parameters (linear and angular) obtained in the lateral teleradiograph of the skull by groups: the *t*-test of independent samples is calculated for this purpose.

	*p*-Value (Test)
Facial axis	0.914
Md plane	0.054
SNA	0.085
SNB	0.647
ANB	0.113
Wits	0.035 *

* *p* < 0.05.

**Table 2:** Homogeneity of cephalometric parameters (linear) obtained in the frontal teleradiograph of the skull by groups: the *t*-test of independent samples is calculated for this purpose.

	*p*-Value (Test)
Craneal width	<0.001 ***
Zyg width	0.067
Orbital width	<0.001 ***
Maxilar width	0.472
Bigonial width	0.694
Biantegonial width	0.189

*** *p* < 0.001.

**Table 3:** Probability of maxillary compression according to each one of the dimensions: Results of independent, unadjusted binary logistic regression models.

	B	E.T.	Wald	gl	*p*-Value	OR	I.C. 95.0% for OR	
							Lower	Upper
**CRANEAL WIDTH**	0.207	0.053	15.251	1	<0.001 ***	1.230	1.109	1.364
**ZYG WIDTH**	0.073	0.040	3.268	1	0.071	1.075	0.994	1.164
**ORBITAL WIDTH**	0.419	0.084	25.179	1	<0.001 ***	1.521	1.291	1.792
**MAXILAR WIDTH**	−0.039	0.054	0.528	1	0.468	0.961	0.864	1.069
**BIGONIAL MD**	0.017	0.043	0.159	1	0.690	1.017	0.935	1.106
**BIANTEGONIAL MD**	0.064	0.049	1.722	1	0.189	1.066	0.969	1.172
**FACIAL AXIS**	0.006	0.053	0.012	1	0.913	1.006	0.907	1.115
**MD PLANE**	0.073	0.038	3.604	1	0.058	1.076	0.998	1.160
**SNA**	−0.118	0.069	2.896	1	0.089	0.889	0.776	1.018
**SNB**	−0.028	0.060	0.215	1	0.643	0.973	0.865	1.094
**ANB**	−0.149	0.095	2.452	1	0.117	0.862	0.715	1.038
**WITTS**	−0.177	0.086	4.231	1	0.040 *	0.838	0.708	0.992

* *p* < 0.05; *** *p* < 0.001.

**Table 4:** Probability of maxillary compression according to the set of dimensions: Results of the multiple binary logistic regression model.

	B	E.T.	Wald	gl	*p*-Value	OR	I.C. 95.0% for OR	
							Lower	Upper
	−47.035	11.13	17.880	1	<0.001 ***	.000		
**CRANEAL WIDTH**	0.171	0.064	7.227	1	0.007 **	1.186	1.047	1.344
**ORBITAL WIDTH**	0.567	0.128	19.675	1	<0.001 ***	1.763	1.372	2.264
**MAXILAR WIDTH**	−0.366	0.105	12.123	1	<0.001 ***	0.693	0.564	0.852

** *p* < 0.01; *** *p* < 0.001.

**Table 5:** Classification table of the subjects on the validation sample, which is represented by 40% of the total sample of patients.

	Total		Control		Compression	
	N	%	N	%	N	%
**Total**	42	100.0%	22	52.4%	20	47.6%
**Control Group**	19	100.0%	17	89.5%	2	10.5%
**Compression Group**	23	100.0%	5	21.7%	18	78.3%

**Table 6:** Diagnostic validity of the current model (estimated on 60% of the sample and estimated on 40%) with respect to the complete one.

	S	E	PPV	PPN	% Correct Total
**Actual model (60−40%)**	89.5%	78.3%	90.0%	77.3%	83.3%
**Complete model (100%)**	88.7%	77.1%	87.3%	79.4%	84.5%
